# Acute cerebellitis in adults: a case report and review of the literature

**DOI:** 10.1186/s13104-017-2935-8

**Published:** 2017-11-22

**Authors:** A. Van Samkar, M. N. F. Poulsen, H. P. Bienfait, R. B. Van Leeuwen

**Affiliations:** 1 Department of Neurology, Canisius-Wilhelmina Ziekenhuis, PO Box 9015, 6500GS Nijmegen, The Netherlands; 2 Department of Radiology, Gelre ziekenhuizen Apeldoorn, PO Box 9014, 7300DS Apeldoorn, The Netherlands; 3 Department of Neurology, Gelre ziekenhuizen Apeldoorn, PO Box 9014, 7300DS Apeldoorn, The Netherlands

**Keywords:** Acute cerebellitis, Acute cerebellar ataxia, Hydrocephalus, Inflammation

## Abstract

**Background:**

Acute cerebellitis is a rare disease with the majority of cases described in children. Little is known about the clinical characteristics and outcome in adults.

**Case presentation:**

A 37-year-old Caucasian woman presented with headache, nausea, and photophobia, and was diagnosed as having a migraine attack. Two days later, she subsequently returned with aggravated headache, dysarthria and horizontal nystagmus. Magnetic resonance imaging (MRI) showed a swollen cerebellum and hydrocephalus and the patient was diagnosed with acute cerebellitis. Cerebrospinal fluid (CSF) examination showed an elevated leukocyte count and protein. Blood serology showed the presence of immunoglobulin M and immunoglobulin G for both Epstein–Barr virus and cytomegalovirus. The patient was treated with dexamethasone and discharged to a rehabilitation center, where she fully recovered. We searched the literature for adult cases of acute cerebellitis. Including our patient, we identified 35 patients with a median age of 36 years. The etiology was unknown in 34% of cases. The most common clinical presentation consisted of headache, nausea/vomiting and ataxia. Six patients presented with only headache and nausea and subsequently returned with cerebellar signs. In 9 cases, the cerebellitis was complicated by hydrocephalus. Half of the patients ended up with neurological sequelae, while follow-up MRI was abnormal in 71%.

**Conclusion:**

Acute cerebellitis in adults is a rare disorder which mainly presents with headache, nausea/vomiting and ataxia. To diagnose cerebellitis, imaging of the brain (preferably MRI) is required and CSF examination may be necessary to narrow the differential diagnosis. The treatment depends on the widely diverse etiology, and treatment with steroids is recommended in the case of cerebellar oedema and hydrocephalus. Neurosurgical intervention may be necessary to prevent brain herniation.

**Electronic supplementary material:**

The online version of this article (10.1186/s13104-017-2935-8) contains supplementary material, which is available to authorized users.

## Background

Acute cerebellitis is a rare inflammatory syndrome. The majority of cases have been described in children and were caused by a primary infection (e.g. West-Nile virus, *Mycoplasma pneumoniae*) or a postinfectious disorder [1]. Acute cerebellitis in children mainly presents with headache and ataxia [2] and may either have a benign, self-limiting course, or present as a fulminant disease requiring neurosurgery and steroid treatment and resulting in severe cerebellar damage [2].

While acute cerebellitis in children has been studied extensively, less is known about acute cerebellitis in adults, due to the small number of cases reported [3]. We describe the case of a 37-year-old woman with acute cerebellitis identified in our hospital and perform a review of the literature on magnetic resonance imaging (MRI)-confirmed acute cerebellitis in adults.

## Case presentation

A 37-year-old Caucasian woman presented on the emergency department with headache, nausea, and photophobia since 2 days. Medical history revealed migraine and an anxiety/panic disorder; family history revealed no abnormalities. Physical examination showed a temperature of 36.3 °C. Neurological examination and computerized axial tomography (CAT)-scan of the brain were normal. The patient was suspected of a migraine attack and dismissed.

**Fig. 1:**
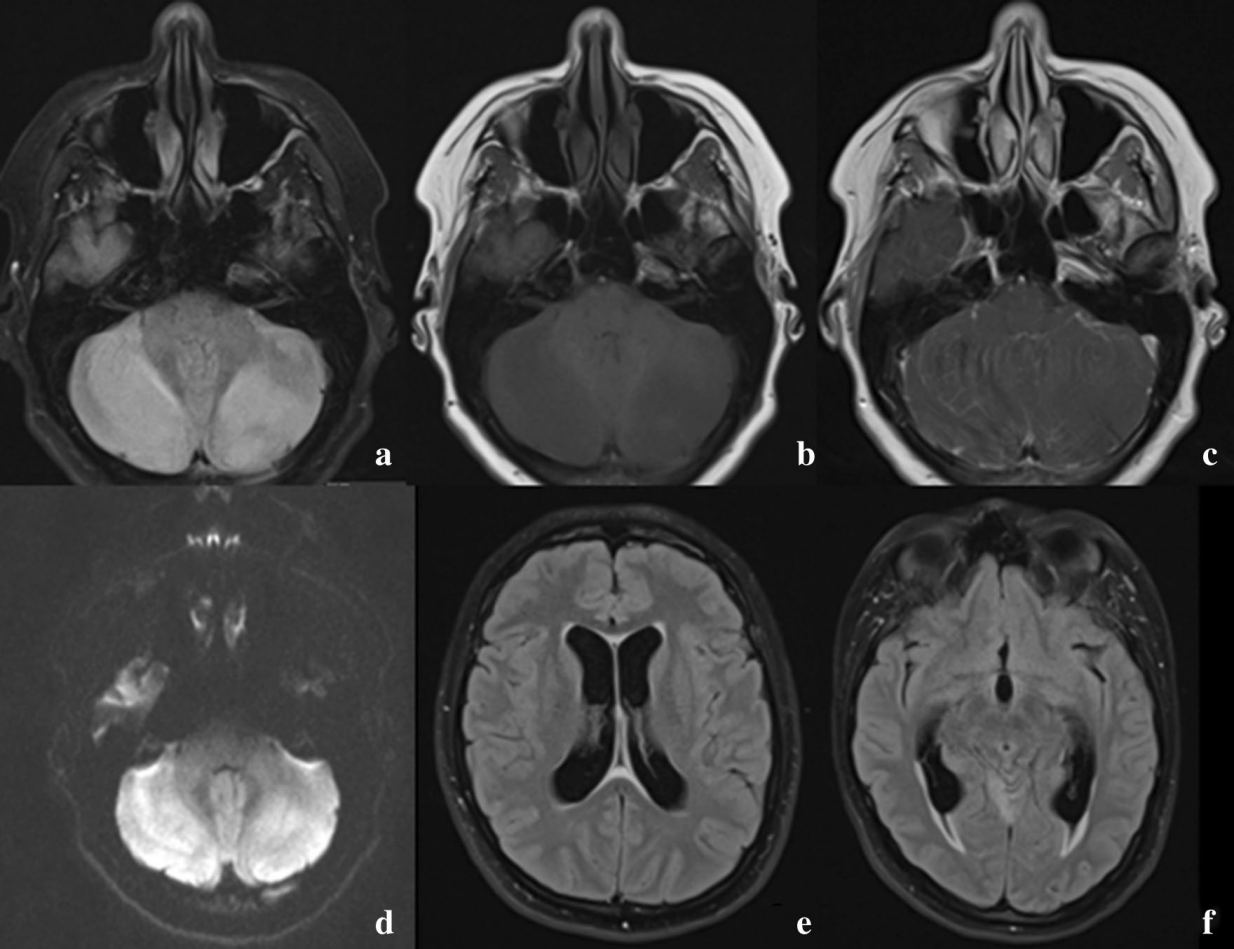
Brain magnetic resonance imaging (MRI) of our case, consistent with cerebellitis and hydrocephalus. From left to right, up to down: **a** FLAIR sequence, showing hyperintense cerebellar hemispheres. **b** T1 sequence, showing hypointense cerebellar hemispheres. **c** T1 sequence with gadolinium, showing leptomeningeal enhancement of the cerebellum. **d** Diffusion sequence, showing restrictive diffusion of the cerebellar hemispheres. **e** FLAIR sequence, showing enlarged lateral ventricles. **f** FLAIR sequence, showing enlarged temporal horns with transependymal oedema, as sign of increased intraventricular pressure

After 2 days, the patient subsequently returned on the emergency department due to aggravated headache, somnolence and slurry speech. Neurological examination showed first degree horizontal nystagmus and dysarthria. The patient was admitted to the neurology ward and an MRI-scan was performed, showing a swollen cerebellum, consistent with cerebellitis, and hydrocephalus (Fig. 1). Lumbar puncture revealed an opening pressure of 28 cmH_2_O, a leukocyte count of 247/mL (92% lymphocytes) and a protein of 1.82 g/L. Treatment with ceftriaxone (2 g twice a day), acyclovir (700 mg three times a day) and dexamethasone (10 mg four times a day) was initiated (Table 1).

**Table 1:** Time line

Day	Event	Diagnostic testing	Treatment
0	Onset of headache, nausea and photophobia	None	None
2	Presentation on the emergency department; diagnosed as migraine	Neurological examination, CAT-scan	None
4	Presentation on the emergency department with dysarthria, nystagmus and aggravated headache	MRI showing swollen cerebellar hemispheres and hydrocephalus	Ceftriaxone, acyclovir, dexamethasone
7	None	Negative cerebrospinal fluid cultures and polymerase chain reactions	Stop ceftriaxone and acyclovir
8	None	–	Stop treatment with dexamethasone
18	Discharge to rehabilitation center	–	–
42	Presentation on outpatient clinic; complete recovery	MRI showing no abnormalities	None

**Fig. 2:**
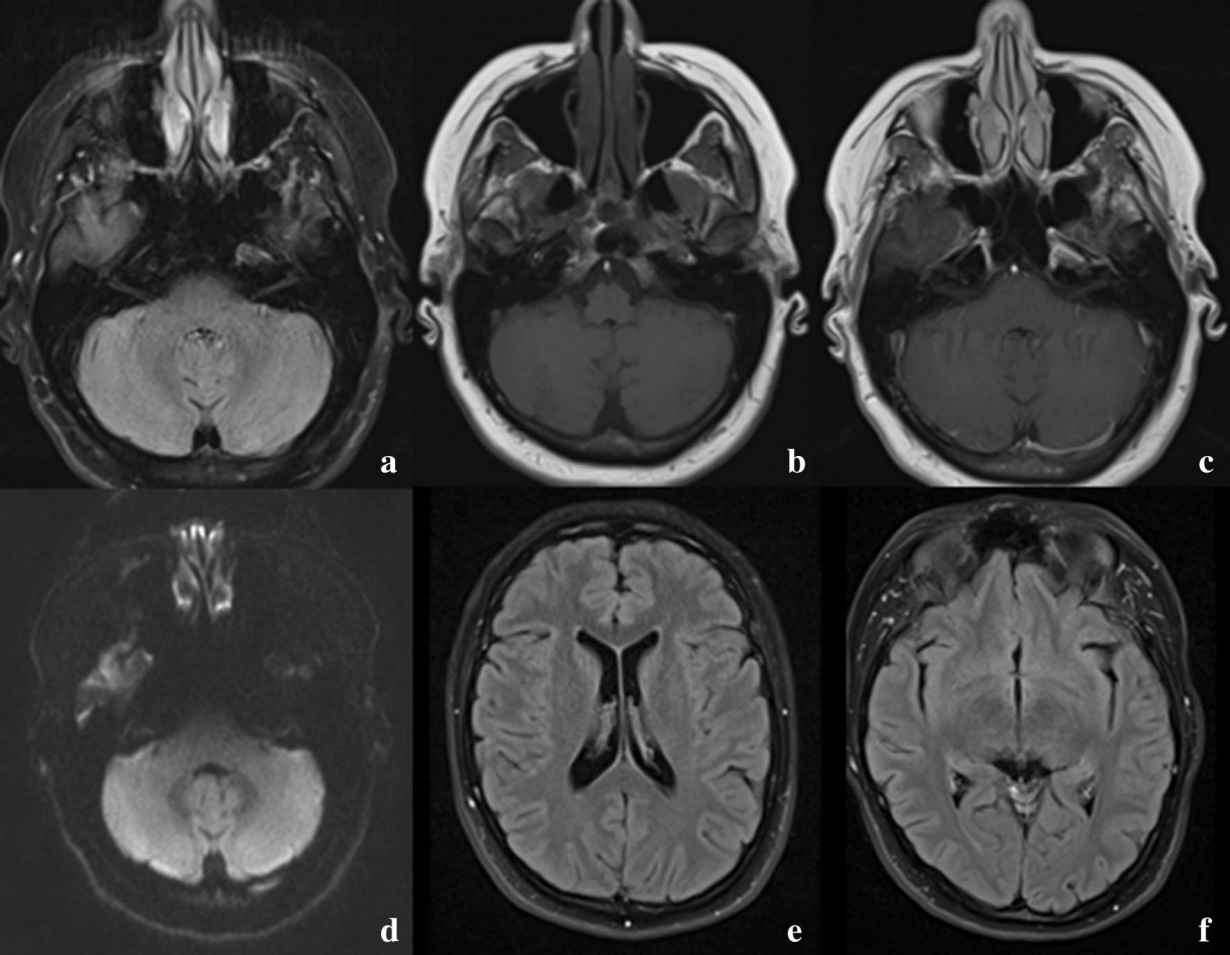
Follow-up magnetic resonance imaging (MRI) scan. From left to right, up to down: **a** FLAIR sequence, showing isointense cerebellar hemispheres. **b** T1 sequence, showing isointense cerebellar hemispheres. **c** T1 sequence with gadolinium, showing no contrast enhancement of the cerebellar hemispheres. **d** Diffusion sequence, showing no restrictive diffusion of the cerebellar hemispheres. **e** FLAIR sequence, showing normalized ventricles. **f** FLAIR sequence, showing normalized temporal horns

Serology, viral polymerase chain reaction (PCR) and cultures of cerebrospinal fluid (CSF) were normal. However, blood serology showed the presence of cytomegalovirus (CMV) immunoglobulin M (IgM) and immunoglobulin G (IgG) and the presence of Epstein–Barr virus (EBV) IgM and IgG. No immune-electrophoresis or assays for auto-immune antibodies were performed. The ceftriaxone and acyclovir were discontinued after 3 days; the dexamethasone was continued for 4 days in total. The patient’s condition improved and after 2 weeks, she was discharged to a rehabilitation centre, where she fully recovered. Neurological examination and follow-up MRI after 6 weeks showed no abnormalities (Fig. 2).

## Discussion and conclusions

Acute cerebellitis in adults is a rare entity with a wide range in etiology, clinical presentation and outcome. The pathophysiology is not fully understood, as the etiology is often unknown, but it may be associated with several pathogens (mostly viruses) and use of medication. In our case, it is possible that either EBV or CMV was the cause, due to CSF lymphocytosis and the presence of both EBV IgM and CMV IgM. However, neither EBV nor CMV could be isolated in the CSF, making a para-infectious phenomenon as reaction to a systemic EBV/CMV infection more likely. Nevertheless, another (unknown) pathogen or an auto-immune cause may play a role.

**Fig. 3:**
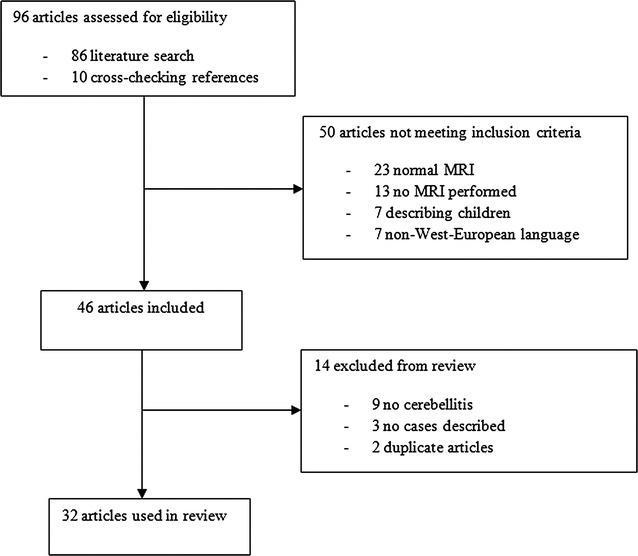
Flowchart review of the literature

A literature search identified 32 studies describing 34 episodes of MRI-confirmed acute cerebellitis in adults, occurring between 1991 and 2016 (Fig. 3). We combined these data with our cases (Table 2, Additional file 1: Table S1), and we identified 35 patients with a median age of 36 years (range 18–73 years). The majority of patients were female (22 out of 35, 63%). More than 80% of patients presented with headache, nausea/vomiting and ataxia. Altered consciousness was reported in 10 out of 35 patients (29%). Six patients, including our case, presented with headache and nausea without other neurological symptoms, and subsequently returned with cerebellar signs.

**Table 2:** Clinical characteristics, etiology and outcome of 35 adult patients with acute cerebellitis, including our case

Characteristics	N/N (%)
Median age (range)	36 (18–73)
Male sex	13/35 (37)
Medical history	
None	6/19 (32)
Malignancy	3/19 (16)
Hepatitis C infection	2/19 (11)
Symptoms	
Headache	23/26 (88)
Fever	12/17 (71)
Nystagmus	13/32 (41)
Vertigo	11/32 (34)
Nausea/vomiting	14/16 (88)
Dysarthria	26/34 (76)
Ataxia	29/31 (94)
Altered consciousness	10/35 (29)
Neck stiffness	4/11 (36)
Etiology	
Unknown	12/35 (34)
Medication-induced	4/35 (11)
Paraneoplastic	3/35 (9)
Para-infectious	2/35 (6)
Epstein–Barr virus in cerebrospinal fluid	2/35 (6)
Influenza in cerebrospinal fluid	2/35 (6)
*Mycoplasma pneumoniae* in cerebrospinal fluid	2/35 (6)
Herpes simplex in cerebrospinal fluid	2/35 (6)
Other	2/35 (6)
Scrub typhus	1/35 (3)
Coxsackie virus in cerebrospinal fluid	1/35 (3)
Salmonella in cerebrospinal fluid	1/35 (3)
Cryptococcus neoformans in cerebrospinal fluid	1/35 (3)
Brain magnetic resonance imaging findings	
T1: cortical hypointensity	7/13 (54)
T2/FLAIR: cortical hyperintensity	23/29 (79)
DWI/ADC: restriction	8/10 (80)
T1 C+ (gadolinium): cortical and leptomeningeal enhancement	18/23 (78)
Hydrocephalus	9/35 (26)
Cerebrospinal fluid findings	
Median leukocyte count (/mL) (range)	104 (0–797)
Median protein (g/L) (range)	0.72 (0.08–2.00)
Treatment	
Steroids	16/35 (46)
Antiviral medication	12/35 (34)
Antibiotics	9/35 (26)
Surgery	7/35 (20)
Outcome	
Full recovery	16/30 (53)
Sequelae	14/30 (47)
Death	0/35 (0)
Follow-up brain magnetic resonance imaging findings	21/35 (60)
Normal	6/21 (29)
Improved, but with persistent abnormalities	11/21 (52)
Cerebellar atrophy	4/21 (19)

The etiology of the cerebellitis was unknown in 12 out of 35 cases (34%) and viral in 8 out of 35 cases (23%). In 2 cases, isoniazid was reported to be the causative agent. Both patients had a history of renal failure, for which they received dialysis. Patients who are on dialysis are more sensitive to isoniazid neurotoxicity, due to pyridoxine deficiency and reduced clearance of isoniazid. Isoniazid neurotoxicity may be prevented by supplementing pyridoxine in patients on dialysis [4, 5].

CSF leukocytes varied widely, from 0 to 797 leukocytes. Brain MRI showed abnormalities on the T1 sequence in about half of the cases (7 out of 13 patients, 54%), while the T2/fluid-attenuated inverse recovery (FLAIR) sequence, diffusion-weighted imaging (DWI)/apparent diffusion coefficient (ADC) sequence and contrast sequence showed abnormalities in about 80% of the cases. Hydrocephalus was seen in 9 out of 35 cases (26%), requiring neurosurgical intervention in 7. Steroids were administered in almost half of the patients (46%, 16 out of 35 patients).

All patients survived, while 14 out of 30 patients (47%) ended up with neurological sequelae, which were mostly persisting cerebellar symptoms such as dysarthria and ataxia.

Follow-up MRI was performed in 21 out of 35 patients (60%), after a period varying between 1 and 24 months. Persisting abnormalities at the time of the follow-up brain MRI were seen in 15 out of 21 patients (71%), consisting of residual changes in 11 patients and cerebellar atrophy in 4 patients.

Patients may present with headache, fever, cerebellar signs and altered consciousness. Six patients, including our case, presented with headache and nausea without other neurological symptoms, and subsequently returned with additional cerebellar signs. Since in our patient the CAT-scan of the brain was normal when the patient presented with headache and nausea, and hydrocephalus was diagnosed 2 days later by MRI, it is hard to diagnose cerebellitis and its complications when a patient primarily presents with headache and nausea without cerebellar signs.

If cerebellar signs are present, the differential diagnosis of cerebellitis is limited and MRI of the brain is needed to confirm the diagnosis and rule out other diagnoses. Alternative diagnoses to be kept in mind are cerebellar stroke, infectious meningoencephalitis, acute disseminated encephalomyelitis, cerebellar tumors—especially when the MRI abnormalities are limited to one cerebellar hemisphere—and posterior reversible encephalopathy syndrome. However, these diseases usually present with different symptoms and thus, it is important to combine the clinical information and radiological information to establish the diagnosis.

CSF examination may help in narrowing the differential diagnosis. However, there is a risk of brain herniation when lumbar puncture is performed in patients with a swollen cerebellum. Therefore, it is advised to perform cranial imaging prior to lumbar puncture. When cranial imaging shows hydrocephalus and compression of the fourth ventricle, lumbar puncture should be discouraged.

The treatment of acute cerebellitis depends on the etiology and complications. When direct invasion by a specific micro-organism is suspected, appropriate antimicrobial and antiviral treatment should be started immediately [6]. When brain MRI shows diffuse cerebellar swelling, patients should also be treated with corticosteroids to prevent further swelling and brain herniation [5]. In the case of secondary hydrocephalus, neurosurgical intervention may be necessary to prevent brain herniation, for example by means of an external ventricle drain [6].

All reported adult patients with acute cerebellitis survived. However, almost half of the patients ended up with neurological sequelae. This is partially explained by cerebellar atrophy, which is a possible complication of acute cerebellitis [7, 8]. In the majority of patients (72%), abnormalities persisted at follow-up MRI, which is probably the result of the cerebellar inflammation which may lead to permanent damage; however, part of these patients did not have any neurological symptoms at all at follow-up MRI.

Our study describes acute cerebellitis in adults. There are several similarities and differences between acute cerebellitis in adults and children. Acute cerebellitis in children generally presented with headache and ataxia [2]. The most common imaging finding at presentation was bilateral diffuse hemispheric involvement, which is similar to acute cerebellitis in adults. However, in children, acute cerebellitis is more often caused by an infectious pathogen, while in adults parainfectious and paraneoplastic causes are more common (Additional file 1: Table S1). Furthermore, pediatric acute cerebellitis generally had a good outcome with full clinical recovery in 50–86% of the cases [2], while in adults half of the patients ended up with neurological sequelae.

A limitation of our study is that abnormal MRI was used as inclusion criterion. In some studies, single photon emission computed tomography (SPECT) is proposed for diagnosis due to its capability to show cerebellar hypoperfusion [9, 10]. However, few studies have been performed comparing SPECT to MRI, without conclusive results, and therefore, MRI remains the gold standard for diagnosing cerebellitis [1, 2, 6].

In conclusion, acute cerebellitis in adults is a rare disorder which mainly presents with headache, nausea/vomiting and ataxia. MRI is the imaging modality of choice and CSF examination may be necessary to narrow the differential diagnosis. The treatment depends on the widely diverse etiology, and treatment with steroids is recommended in the case of cerebellar oedema and hydrocephalus. Neurosurgical intervention may be necessary to prevent brain herniation.

## Additional file

**Additional file 1: Table S1.** Clinical characteristics, etiology and outcome of 35 adult patients with acute cerebellitis confirmed by MRI. This file contains a spreadsheet of the data extracted from the articles describing patients with MRI-confirmed acute cerebellitis.

## Abbreviations

ADC: apparent diffusion coefficient
CAT: computerized axial tomography
CMV: cytomegalovirus
CSF: cerebrospinal fluid
DWI: diffusion-weighted imaging
EBV: Epstein–Barr virus
FLAIR: fluid-attenuated inverse recovery
IgG: immunoglobulin G
IgM: immunoglobulin M
MRI: magnetic resonance imaging
PCR: polymerase chain reaction
SPECT: single photon emission computed tomography
