# Supplementary material for: Acute cerebellitis in adults: a case report and review of the literature
Source: BMC Res Notes. 2017 Nov 22;10:610. doi: 10.1186/s13104-017-2935-8 (PMC5700531; doi:10.1186/s13104-017-2935-8)
Supplement: Supplementary file 1 — Additional file 1: Table S1. Clinical characteristics, etiology and outcome of 35 adult patients with acute cerebellitis confirmed by MRI. This file contains a spreadsheet of the data extracted from the articles describing patients with MRI-confirmed acute cerebellitis. [file 13104_2017_2935_MOESM1_ESM.doc]

**Table S1. Clinical characteristics, etiology and outcome of 35 adult patients with acute cerebellitis confirmed by MRI**

| **Author** | **Age** | **Gender** | **Medical history** | **Etiology** | **Symptoms** | **CSF leukocytes** | **Other MRI findings** | **Outcome** |
| --- | --- | --- | --- | --- | --- | --- | --- | --- |
| **Our case*** | 37 | F | Migraine, anxiety disorder | CMV, EBV | 1,2,3,5,6,8 | 247 | Hydrocephalus | Full recovery |
| **Chaitanya[11]** | 53 | M | Chronic renal failure | Isoniazid | 7 | Not performed | None | Unknown |
| **Bhoil et al[12]** | 21 | M | Unknown | Scrub typhus | 2,6,7,8 | Unknown | None | Unknown |
| **Martins et al[13]** | 19 | F | Unknown | Recent RTI | 1,5,7 | Unknown | None | Full recovery |
| **Lee et al[7]** | 35 | M | Unknown | Castleman | 4,6,7 | 16 | None | Wheelchair dependent |
| **Hamaji et al[14]** | 30 | M | Unknown | Castleman | 4,6,7 | Unknown | None | Persisting symptoms |
| **Gupta et al[15]** | 73 | M | Lymphocytic lymphoma | Unknown | 1,3,5,7 | 1 | Hydrocephalus | Persisting symptoms |
| **Liu et al[16]** | 31 | M | Unknown | EBV | 1,5,6,7 | Elevated | Hydrocephalus | Full recovery |
| **Sfeir et al[17]** | 37 | F | Unknown | Influenza | 1,2,5,6,7 | 330 | None | Persisting symptoms |
| **Choi* et al[18]** | 59 | F | Unknown | Unknown | 1,3,4,5,7 | 180 | None | Persisting symptoms |
|  | 25 | F | Unknown | Unknown | 1,3,4,7,9 | 227 | None | Full recovery |
| **Peter et al[19]** | 40 | F | Chronic renal failure | Isoniazid | 4,6,7 | Normal | None | Full recovery |
| **Flanagan et al[20]** | 41 | M | Crohn’s disease | Crohn | 1,4,5,6,7 | Normal | Hydrocephalus | Unknown |
| **Patel et al[3]** | 61 | F | Alcohol abuse | Unknown | 2,6,7,8 | Not performed | None | Wheelchair dependent |
| **Rizek et al[21]** | 63 | F | Diabetes mellitus | Salmonella | 6,7,8 | 15 | None | Persisting dysarthria |
| **De Santis et al[22]** | 47 | M | Hepatitis C | Influenza | 1,5,8 | 0 | Hydrocephalus | Persisting comatose state |
| **Hassan et al[23]** | 19 | F | Unknown | HSV | 2,8 | 70 | None | Full recovery |
| **Marignier et al[8]** | 50 | F | None | Anti-mGluR1 | 3,6,7 | 190 | None | Persisting ataxia |
| **Morales et al[24]** | 46 | M | Hepatitis C, drug abuse | Opioids | 6,7 | 1 | Hydrocephalus | Persisting ataxia and dysarthria |
| **Karmon* et al[6]** | 29 | M | Unknown | Hodgkin | 1,3,6,7 | 270 | None | Persisting ataxia |
| **Fickweiler et al[25]** | 61 | F | Multiple myeloma | Cryptococcus | 3,4,5,7 | Unknown | Hydrocephalus | Full recovery |
| **Hashimoto et al[26]** | 58 | F | None | EBV | 1,2,6,7 | 797 | None | Full recovery |
| **Donmez et al[27]** | 35 | F | None | Unknown | 1,2,4,5,6,7,9 | 140 | None | Full recovery |
| **Bielsa-Martin et al[28]** | 38 | F | Unknown | Mycoplasma | 1,6,7 | 195 | None | Full recovery |
| **De Bruecker et al[1]** | 26 | F | Unknown | Unknown | 1,2,3,6,7,8 | 138 | None | Persisting cerebellar signs |
| **Riverol et al[29]** | 18 | F | None | Coxsackie | 1,2,6 | Elevated | None | Unknown |
| **Gruis* et al[30]** | 38 | F | Unknown | Unknown | 1,3,4,5,6,7 | 62 | None | Full recovery |
| **Guerrini et al[9]** | 36 | M | Unknown | Unknown | 1,2,6,7 | 6 | None | Full recovery |
|  | 19 | M | Unknown | Mycoplasma | 1,6 | Unknown | None | Full recovery |
| **Ciardi et al[31]** | 24 | F | Unknown | HSV | 1,2,3,5,6,7 | 20 | None | Full recovery |
| **Yeshurun et al[32]** | 64 | F | Lymphoma | Cytarabine | 3,6,7 | Unknown | None | Persisting symptoms |
| **Sugiyama* et al[33]** | 35 | F | None | Unknown | 1,2,3,4,6,8,9 | 205 | None | Full recovery |
| **Ravi et al[34]** | 22 | F | Sickle cell disease | Unknown | 1,2,5,7,9 | Elevated | Hydrocephalus | Unknown |
| **Bakshi et al[35]** | 21 | M | None | Unknown | 1,5,6,7 | 17 | Hydrocephalus | Persisting dysarthria |
| **Shoji et al[36]** | 24 | F | Unknown | Unknown | 1,3,6,7,8 | 45 | None | Full recovery |

Abbreviations: M: male; F: female; HSV: herpes simplex virus; CMV: cytomegalovirus; EBV: Epstein Barr virus; RTI: respiratory tract infection. Symptoms: 1: headache; 2: fever; 3: nystagmus; 4: vertigo; 5: nausea/vomiting; 6: dysarthria; 7: ataxia; 8: altered consciousness, 9: neck stiffness. *Patients first presenting with headache and nausea, with secondary cerebellar symptoms

**Additional references**

11. Chaitanya V, Sangeetha B, Reddy MH, Venkata Kumar AC, Ram R, Sivakumar V. Isoniazid cerebellitis in a peritoneal dialysis patient. *Nephrology (Carlton).* 2016; 21:442.

12. Bhoil R, Kumar S, Sood RG, Bhoil S, Verma R, Thakur R. Cerebellitis as an atypical manifestation of scrub typhus. *Neurology.* 2016; 86:2113-4.

13. Martins WA, Cristovam Rdo A, Palmini A. Acute post-infectious cerebellitis. *Arq Neuropsiquiatr.* 2015; 73:977.

14. Hamaji M, Neal JW, Burt BM. Unicentric, multifocal Castleman disease of the mediastinum associated with cerebellitis. *Ann Thorac Surg.* 2015; 99:e7-9.

15. Gupta R, Maralani PJ, Chawla S, Gopal PP, Mohan S. Advanced neuroimaging findings of pseudotumoral hemicerebellitis in an elderly male requiring surgical decompression. *J Neurosurg.* 2014; 120:522-7.

16. Liu WC, Chiu SK, Hsiang CW, Lin TY. Acute unilateral cerebellitis, Epstein-Barr virus, and HIV. *Lancet Infect Dis.* 2014; 14:778.

17. Sfeir MM, Najem CE. Cerebellitis associated with influenza A(H1N1)pdm09, United States, 2013. *Emerg Infect Dis.* 2014; 20:1578-80.

18. Choi JY, Kim JS, Jung JM, Kwon DY, Park MH, Kim C, Choi J. Reversed corrective saccades during head impulse test in acute cerebellar dysfunction. *Cerebellum.* 2014; 13:243-7.

19. Peter P, John M. Isoniazid-induced cerebellitis: a disguised presentation. *Singapore Med J.* 2014; 55:e17-9.

20. Flanagan EP, Rabinstein AA, Kumar N, Schroeder K, Kantarci OH. Fulminant cerebellitis with radiological recurrence in an adult patient with Crohn's disease. *J Neurol Sci.* 2014; 336:247-50.

21. Rizek P, Morriello F, Sharma M, Gofton T. Teaching NeuroImages: acute cerebellitis caused by Salmonella typhimurium. *Neurology.* 2013; 80:e118.

22. De Santis P, Della Marca G, Di Lella G, Cavallaro F. Neurological picture. Sub-acute hydrocephalus in a patient with influenza A (H3N2) virus-related cerebellitis. *J Neurol Neurosurg Psychiatry.* 2012; 83:1091-2.

23. Hassan H, Thomas B, Iyer RS. Insights from a rare clinical presentation of herpes simplex encephalitis: adding to the catatonic dilemma? *Neurologist.* 2011; 17:114-6.

24. Morales Odia Y, Jinka M, Ziai WC. Severe leukoencephalopathy following acute oxycodone intoxication. *Neurocrit Care.* 2010; 13:93-7.

25. Fickweiler W, Aries MJ, Enting RH, Vellenga E, De Keyser J. Cryptococcal cerebellitis after chemotherapy and autologous stem cell re-infusion in a patient with multiple myeloma. *J Neurol.* 2009; 256:145-6.

26. Hashimoto Y, Kobayashi Z, Kotera M. Leptomeningeal enhancement in acute cerebellitis associated with Epstein-Barr virus. *Intern Med.* 2008; 47:331-2.

27. Donmez FY, Agildere AM, Tore HG, Ure S, Benli S. Abnormal diffusion-weighted imaging findings in an adult patient with acute cerebellitis presenting with a normal magnetic resonance imaging. *J Comput Assist Tomogr.* 2008; 32:156-8.

28. Bielsa-Martin S, Porcel-Perez JM, Madronero-Vuelta AB. [Acute cerebellitis caused by Mycoplasma pneumoniae]. *Rev Neurol.* 2005; 41:128.

29. Riverol M, Irimia P, Martinez-Vila E. Cerebellitis. *Lancet.* 2003; 362:1449.

30. Gruis KL, Moretti P, Gebarski SS, Mikol DD. Cerebellitis in an adult with abnormal magnetic resonance imaging findings prior to the onset of ataxia. *Arch Neurol.* 2003; 60:877-80.

31. Ciardi M, Giacchetti G, Fedele CG, Tenorio A, Brandi A, Libertone R, Ajassa C, Borgese L, Delia S. Acute cerebellitis caused by herpes simplex virus type 1. *Clin Infect Dis.* 2003; 36:e50-4.

32. Yeshurun M, Marsot Dupuch K. Acute cerebellar syndrome following intermediate-dose cytarabine. *Br J Haematol.* 2001; 113:846.

33. Sugiyama N, Hamano S, Mochizuki M, Tanaka M, Takahashi Y. [A case of chronic cerebellitis with anti-glutamate receptor delta 2 antibody]. *No To Hattatsu.* 2004; 36:60-3.

34. Ravi V, Rozen TD. Acute cerebellitis: MRI findings. *Neurology.* 2000; 54:213.

35. Bakshi R, Bates VE, Kinkel PR, Mechtler LL, Kinkel WR. Magnetic resonance imaging findings in acute cerebellitis. *Clin Imaging.* 1998; 22:79-85.

36. Shoji H, Goto Y, Yanase Y, Sato Y, Nakashima K, Natori H, Kaji M. Recurrent cerebellitis. A case report of a possible relationship with Epstein-Barr infection. *Kurume Med J.* 1983; 30:23-6.
